# Comparative Transcriptome Combined with Morphophysiological Analyses Revealed Carotenoid Biosynthesis for Differential Chilling Tolerance in Two Contrasting Rice (*Oryza sativa* L.) Genotypes

**DOI:** 10.1186/s12284-023-00669-6

**Published:** 2023-11-25

**Authors:** Peng Zhang, Xiang Wu, Yulin Chen, Guangmei Ji, Xinling Ma, Yuping Zhang, Jing Xiang, Yaliang Wang, Zhigang Wang, Liangtao Li, Huizhe Chen, Yikai Zhang

**Affiliations:** 1 State Key Laboratory of Rice Biology and Breeding, China National Rice Research Institute, Hangzhou, 310006 Zhejiang People’s Republic of China; 2 College of Landscape and Ecological Engineering, Hebei University of Engineering, Handan, 056009 Hebei People’s Republic of China; 3 Guizhou Rice Research Institute, Guiyang, 550009 Guizhou People’s Republic of China

**Keywords:** Rice, Carotenoid biosynthesis, Chloroplast development, Cold tolerance, Greening process

## Abstract

Early spring cold spells can lead to leaf chlorosis during the rice seedling greening process. However, the physiological and molecular mechanisms underlying the rice greening process under low-temperature conditions remain unknown. In this study, comparative transcriptome and morphophysiological analyses were performed to investigate the mechanisms mediating the responses of the Koshihikari (Kos) and Kasalath (Kas) rice cultivars to chilling stress. According to their growth-related traits, electrolyte leakage, and chlorophyll fluorescence parameters, Kos was more tolerant to low-temperature stress than Kas. Moreover, chloroplast morphology was more normal (e.g., oval) in Kos than in Kas at 17 °C. The comparative transcriptome analysis revealed 610 up-regulated differentially expressed genes that were common to all four comparisons. Furthermore, carotenoid biosynthesis was identified as a critical pathway for the Kos response to chilling stress. The genes in the carotenoid biosynthesis pathway were expressed at higher levels in Kos than in Kas at 17 °C, which was in accordance with the higher leaf carotenoid content in Kos than in Kas. The lycopene *β*-cyclase and lycopene *ε*-cyclase activities increased more in Kos than in Kas. Additionally, the increases in the violaxanthin de-epoxidase and carotenoid hydroxylase activities in Kos seedlings resulted in the accumulation of zeaxanthin and lutein and mitigated the effects of chilling stress on chloroplasts. These findings have clarified the molecular mechanisms underlying the chilling tolerance of rice seedlings during the greening process.

**Supplementary Information:**

The online version contains supplementary material available at 10.1186/s12284-023-00669-6.

## Introduction

In early spring, rice seedlings emerging from the soil are susceptible to cold stress, which increases the likelihood of frost damage and disease (Andaya and Tai 2007). The newly developed tray-overlaying seedling raising mode for transplanting rice addresses the problems of uneven emergence, rotten seedlings, and serious diseases caused by cold stress in early spring (Zhu et al. 2018). However, during the transfer of rice seedlings from a warm (i.e., temperature controlled) dark room to cold conditions outdoors, some seedlings remain susceptible to low-temperature stress in early spring, resulting in leaf chlorosis (Yoshida et al. 1996; Fukuda et al. 2021).

Plant responses to low temperatures are mediated by a complex mechanism involving Ca^2+^ signaling, reactive oxygen species (ROS) homeostasis, phytohormones, and MAPK signaling (Das and Roychoudhury 2014; Wang et al. 2021a). Plants produce a variety of antioxidants to counteract the oxidative stress induced by low temperatures, including carotenoids, tocopherols, ascorbic acid, glutathione, and antioxidant enzymes (Yun et al. 2010; Havaux 2014). Additionally, when seedlings kept in darkness are exposed to light, their incompletely developed chloroplasts cannot process the sudden increase in light, which leads to photo-oxidative stress (op den Camp et al. 2003; Hideg et al. 2006). During this process, carotenoids, which function as antioxidants, also serve as pigments that protect chloroplasts from photo-oxidative stress exacerbated by low temperatures (Nisar et al. 2015; Dhami and Cazzonelli 2020; Maslova et al. 2021; Sun et al. 2022). The significant correlations among low-temperature stress, carotenoid biosynthesis pathways, and phytohormone signaling have been investigated in many plants (Dhami and Cazzonelli 2020; Li et al. 2021; Yang et al. 2021). A transcriptome-based acclimatization analysis of the photo/thermo-sensitive genic male sterile rice line Yu17S indicated that carotenoid biosynthesis and phytohormone signaling are closely related to cold tolerance (Pan et al. 2022).

The carotenoid biosynthesis pathway is initiated following the synthesis of geranylgeranyl pyrophosphate through the MEP pathway. Phytoene synthase (PSY), which is the primary rate-limiting enzyme of the carotenoid biosynthesis pathway, catalyzes the synthesis of 15-cis-phytoene (Gao et al. 2010; Ruiz-Sola and Rodriguez-Concepcion 2012). Subsequently, all-trans-lycopene is synthesized from 15-cis-phytoene via reactions catalyzed by phytoene desaturase, *ζ*-carotene isomerase, *ζ*-carotene desaturase, and phytoene desaturase. Next, *β*- and *α*-carotene are synthesized by the cyclization of all-trans-lycopene mediated by lycopene *ε*-cyclase (LCYE) and lycopene *β*-cyclase (LCYB), respectively. Previous research indicated that LCYE and LCYB control a major branch of the carotenoid biosynthesis pathway (Ruiz-Sola and Rodriguez-Concepcion 2012; Moreno et al. 2016; Kang et al. 2018). Four carotenoid hydroxylases (CHs), which are classified as *β*-ring hydroxylases (BCHs) and cytochrome P450-type hydroxylases (CYP97A/CYP97C), hydroxylate *α*- and *β*-carotene to produce lutein and zeaxanthin. In addition, *β*-carotene and lutein, which are key components of photosynthetic membranes, form a pigment–protein complex essential for photoprotection (Jahns and Holzwarth 2012; Leonelli et al. 2017; Ding et al. 2021). The production of zeaxanthin is catalyzed by zeaxanthin epoxidase (ZEP) and reversed by violaxanthin de-epoxidase (VDE). This process, known as the xanthophyll cycle, is one of the primary mechanisms protecting plants from the detrimental effects of excessive light (Gao et al. 2010; Wang et al. 2021b). Abscisic acid synthesis, which is catalyzed by 9-cis-epoxycarotenoid dioxygenase (NCED), is considered to be crucial for carotenoid degradation.

Rice (*Oryza sativa* L.) is a thermophilic crop (optimal growth temperature: 20–33 °C) in the family *Poaceae* and is a major contributor to global food security. There has recently been an increase in the frequency of abnormally low temperatures in early spring (Gao et al. 2022; Zhang and Wang 2022). Leaf chlorosis is caused by the inhibition of chlorophyll biosynthesis and oxidative damage when rice seedlings are exposed to cold conditions (Zhao et al. 2020; Fukuda et al. 2021). Moreover, the seedlings of some rice cultivars do not turn green after they are transplanted from a darkened chamber in which the temperature is controlled to a field with a lower temperature. In a recent study involving the *indica* rice cultivar Kasalath, chlorotic leaves were detected on plants kept at low temperatures, indicative of the sensitivity of this variety to cold stress (Fukuda et al. 2021). In contrast, Koshihikari is a cold-tolerant rice variety (Gazquez et al. 2020; Rayee et al. 2020). Therefore, these two rice varieties were selected as appropriate materials for studying the physiological and molecular mechanisms against chilling stress. Previous analyses of the chilling tolerance of these two rice varieties focused on physiological factors, such as photosynthetic capacity (Gazquez et al. 2018), water use efficiency (Gazquez et al. 2015), and secondary metabolism (Fukuda et al. 2021). Accordingly, the morphophysiological and molecular mechanisms that explain the difference in the cold tolerance of these two rice varieties should be examined in more detail. In this study, Kasalath (Kas) and Koshihikari (Kos) were compared in terms of their cold tolerance, chloroplast structure, and chlorophyll fluorescence. Furthermore, a comparative transcriptome analysis was carried out to investigate the molecular mechanisms responsible for cold tolerance. Our results provide further evidence of the importance of carotenoid biosynthesis in the leaf greening process of *Poaceae* plants exposed to low temperatures.

## Results

### Kos Exhibited Higher Tolerance to Low-Temperature Stress

**Fig. 1:**
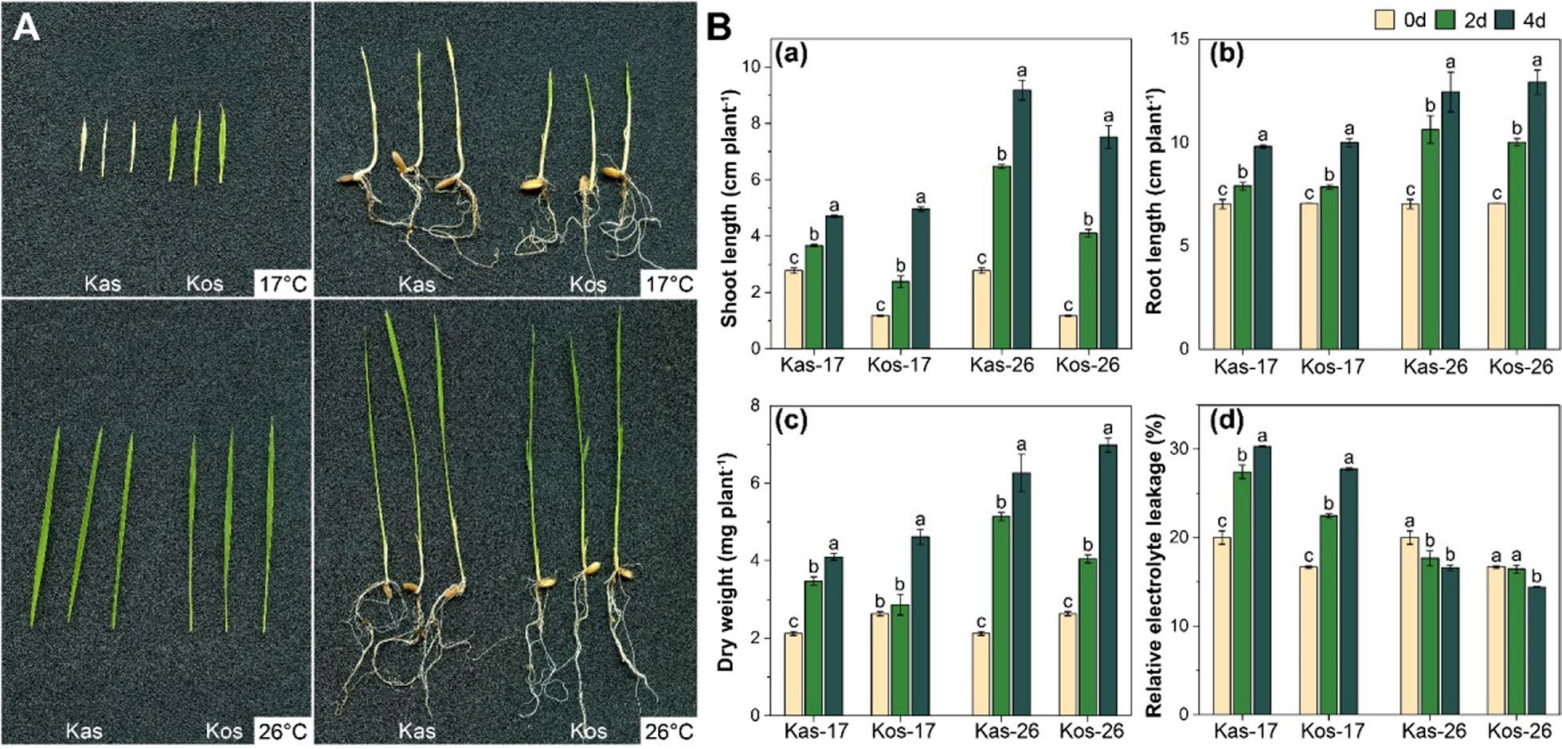
The seedling growth of two rice genotypes at 17 and 26 °C during greening process. **A** The seedling of two rice genotypes. Kas-17, Kos-17, Kas-26, and Kos-26 indicate treatments of Kas and Kos at 17 and 26 °C, respectively, same as afterwards. The pictures were taken on day 4. **B** Shoot length, root length, dry weight, and relative electrolyte leakage of two genotypes seedlings. Different letters above the bars represent significant differences at *p* < 0.05, same as afterwards. Values are means ± SD (three independent biological replicates and four plants for each biological replicate), same as afterwards

The phenotypes of Kas and Kos were compared at 17 and 26 °C (Fig. 1A). The Kas and Kos seedlings grew normally and turned green at 26 °C. However, at 17 °C, the Kos seedlings turned green, but the Kas leaves appeared yellowish-white and yellowish-green during the greening process. These results suggested the greening process was slower for Kas than for Kos under chilling stress.

The shoot length, root length, dry weight and relative electrolyte leakage (REL), also differed between the two cultivars at 17 and 26 °C (Fig. 1B). The trait values of both cultivars were lower at 17 °C than at 26 °C, especially the shoot length. Among the examined traits, the shoot length increased by 229.70% (Kas) and 536.72% (Kos) on day 4 (compared with the shoot length on day 0) at 26 °C. However, at 17 °C, the shoot length exhibited a smaller increase of only 58.98% (Kas) and 320.62% (Kos) on day 4 (compared with the shoot length on day 0). This led to a more pronounced decrease in shoot length for Kas than for Kos under low temperature. Additionally, REL increased for both cultivars under low temperature. At 17 °C, REL was higher for Kas than for Kos, indicating that Kos exhibited superior tolerance to chilling stress compared to Kas.

**Fig. 2:**
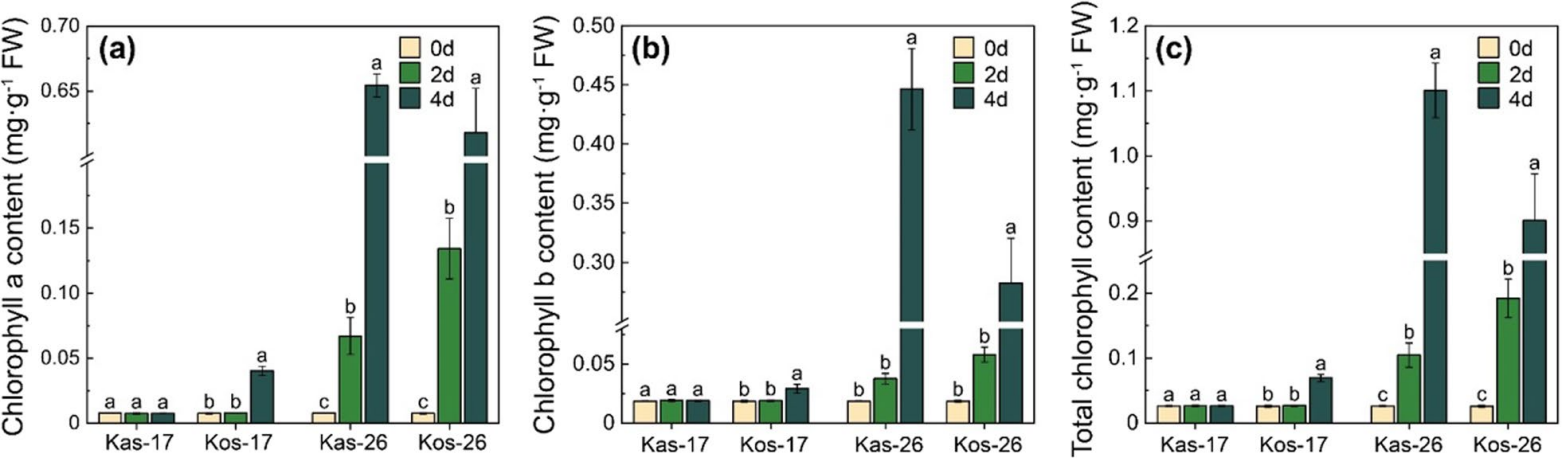
Chlorophyll content of two rice genotypes at 17 and 26 °C during greening process. **a** Chlorophyll *a* content. **b** Chlorophyll *b* content. **c** The total chlorophyll content. Values are means ± SD (three independent biological replicates and four plants for each biological replicate). Different letters above the bars represent significant differences at *p* < 0.05, same as afterwards

The chlorophyll and carotenoid contents of the treated seedlings were measured (Fig. 2). Compared with Kas, the chlorophyll a and b content at 17 °C were significantly higher in Kos on day 4 (5.33- and 1.54-folds higher, respectively), whereas showed lower in Kos on day 4 at 26 °C (1.06- and 1.59-folds lower, respectively). The data indicated that the contents of chlorophyll *a* and *b* were increased in Kos (compared with those in Kas) on day 4 at 17 °C (Table 1). These results reflected a higher chlorophyll biosynthesis in Kas in response to low temperature.

**Table 1:** Results of three-way ANOVA interaction (Temperature × Genotype × Day, followed with Duncan test) and main effects

Indicators	F value of three-way ANOVA			
	Temperature (T)	Genotype (G)	Day (D)	T×G×D
Shoot length	702***	358.5***	1338.9***	14.7***
Root length	111.9***	0^NS^	221.3***	0.6^NS^
Dry weight	254.2***	1.4^NS^	542.2***	1.8^NS^
Chlorophyll a	2970.5***	6*	2176.3***	20.7***
Chlorophyll b	605.2***	19.8***	506***	38***
Total chlorophyll	1688.6***	1.8^NS^	1294.9***	32.2***
REL	1275.1***	215.1***	134.6***	9***
Violaxanthin	5258.2***	179***	14089.9***	441.2***
Zeaxanthin	7499.4***	232.5***	14,764***	375.9***
Lutein	3186***	105.7***	6786.7***	172.8***
*β*-carotenoid	262.9***	57.3***	688.4***	13.1***
NCED	91.9***	15.4***	613.2***	22***
CH	153***	134.3***	353.3***	9.2***
LYCE	270***	50.2***	1006.2***	79.1***
LCYB	1148***	246.5***	546.8***	839.2***
VDE	529.1***	47.7***	1967.6***	200.1***
CCD	247***	118.5***	1207.2***	42.8***
D27s	17.4***	1517.7***	1281.5***	22.6***
PSY	16.8***	4.8*	204.2***	0.6^NS^

*NS* Not significant

* and *** indicate significant differences at *p* < 0.05 and 0.001 probability levels, respectively

### Kos Presented a Better-Maintained Photosystem Morphological Structure Under Chilling Stress

**Fig. 3:**
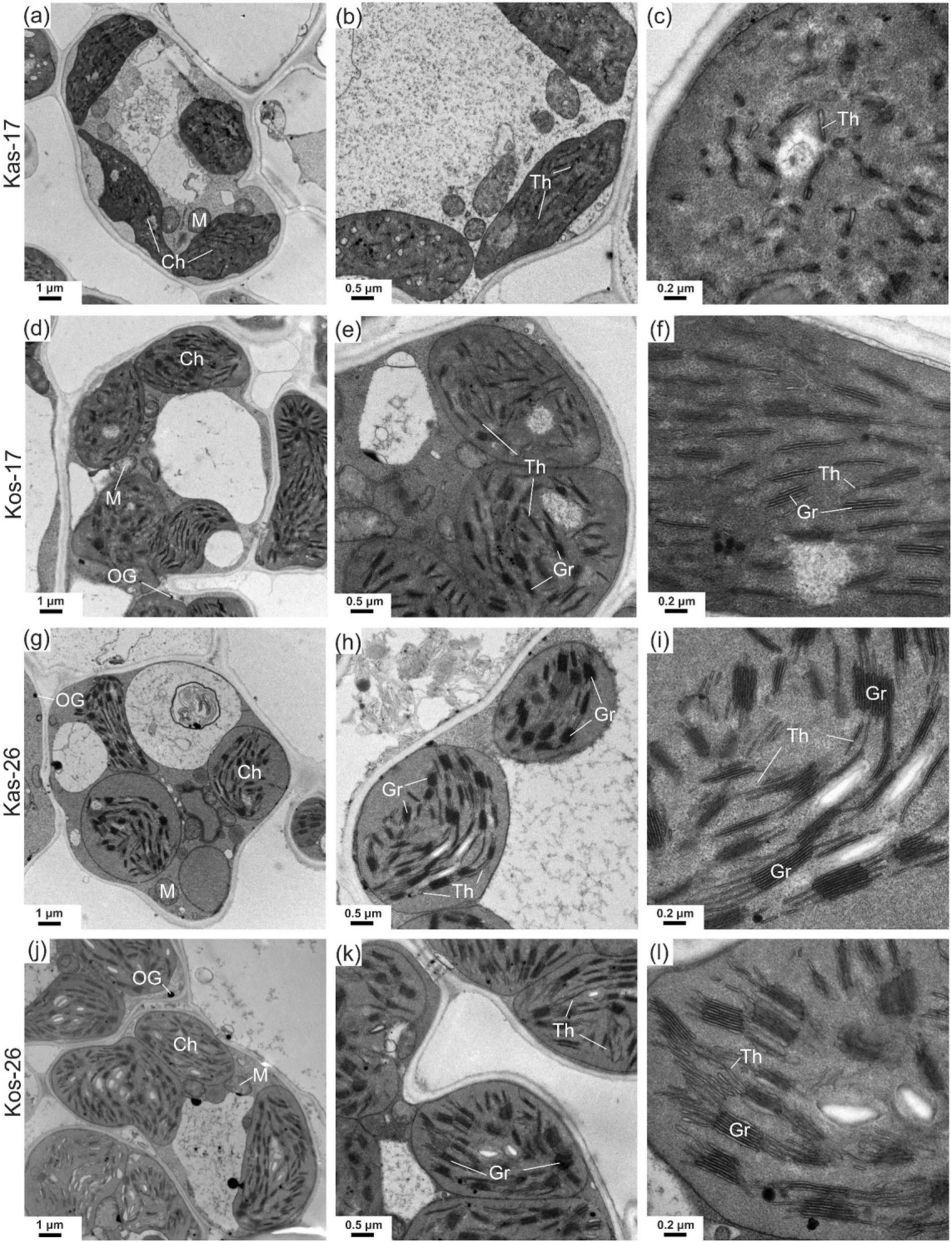
The chloroplast ultrastructure in seedling leaves of two rice genotypes at 17 and 26 °C during greening process. **a**–**c** Kas-17 (17 °C, 4d). **d**–**f** Kos-17 (17 °C, 4d). **g**–**i** Kas-26 (26 °C, 4d). **j**–**l** Kos-26 (26 °C, 4d). *Ch* Chloroplast, *M* Mitochondria, *Th* Thylakoid grana, *Gr* Grana, *OG* Osmium granule

The chloroplast ultrastructure of the rice seedlings was observed to clarify the effect of chilling stress (Fig. 3). Chloroplast development was inhibited in both cultivars at 17 °C, resulting in irregular chloroplast morphology and uneven stacking of cystoid stroma lamellae. The chloroplasts of Kos had more thylakoids stacked into grana than the chloroplasts of Kas. In terms of morphology, the chloroplasts in Kos were more normal (i.e., oval) than the chloroplasts in Kas at 17 °C. Hence, the abnormal development of the chloroplasts and thylakoid membrane structures were the dominant factors explaining the leaf chlorosis of the Kas seedlings exposed to chilling stress.

**Fig. 4:**
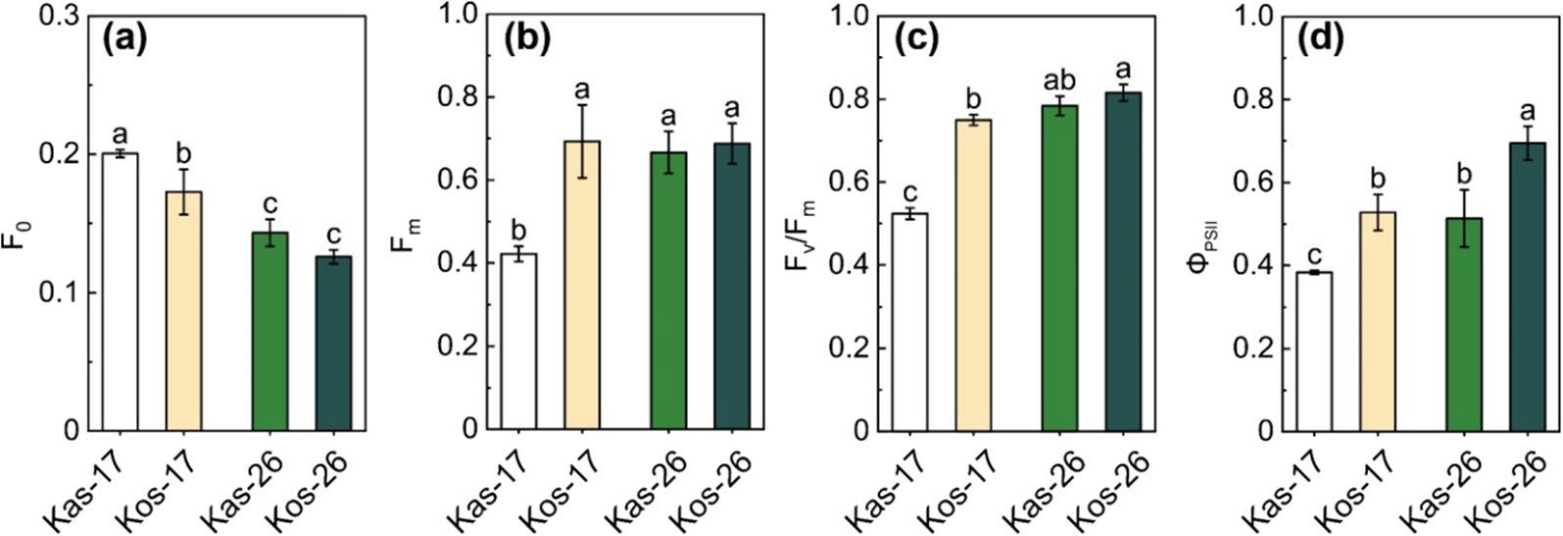
The fluorescence parameters in seedling leaves of two genotypes at 17 and 26 °C during greening process. **a** F_0_. **b** F_v_/F_m_. **c** F_m_. **d** Φ_PSII_. Values are means ± SD (three independent biological replicates for each sample and three technical replicates for each biological replicate). Different letters above the bars represent significant differences at *p* < 0.05

A Pulse amplitude modulation (PAM) chlorophyll fluorescence meter was used in this study to investigate whether leaf photosynthetic activities were affected by the low-temperature treatment. The maximum (F_v_/F_m_) and actual photosynthetic capacity of PSII (Φ_PSII_) of both cultivars were significantly lower at 17 °C than at 26 °C (Fig. 4). More specifically, the F_v_/F_m_ value of Kas at 26 °C was 1.43- folds higher than those at 17 °C, whereas in Kos, it was only 1.04- folds higher than those at 17 °C. Moreover, F_v_/F_m_ was significantly lower for Kas than for Kos at 17 °C, but there was no significant difference between Kas and Kos at 26 °C (Fig. 4). These results suggested the photosystem morphological structure and the PSII reaction center energy conversion efficiency maintained better in Kos than in Kas exposed to chilling stress.

### Carotenoid Biosynthesis was Identified as a Key Pathway by the Comparative Transcriptome Analysis of the Leaves Under Chilling Stress Conditions

To further investigate the molecular mechanisms underlying the differences in the greening process of the two rice genotypes that underwent the low-temperature treatment, we conducted a comparative transcriptome analysis using the high-throughput sequencing system. During the temperature treatments (17 and 26 °C), a total of 30 libraries were constructed for the two examined rice genotypes (Kas and Kos). This period is crucial for determining whether rice seedlings can withstand the effects of cold stress. After removing the low-quality reads, more than 40,000,000 clean reads were retained for each sample. The transcriptome sequencing profiles for each sample are provided in Additional file 1: Table S2.

**Fig. 5:**
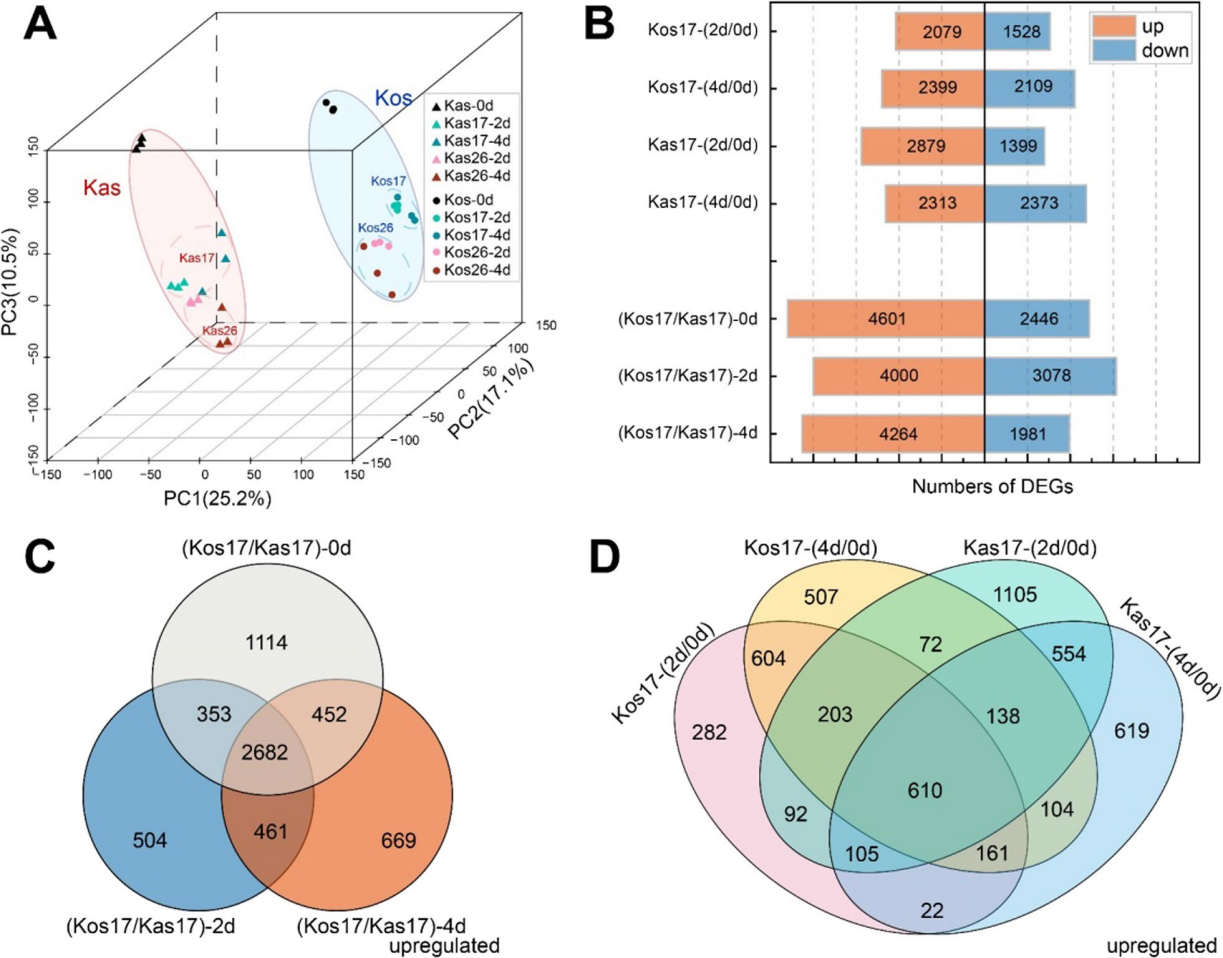
Principal component analysis (PCA) results and numbers of DEGs in two genotypes during greening. **A** Distribution of Kas and Kos in PC1, PC2, and PC3, with the percentage variance of each component. **B** Number of DEGs in Kas and Kos at 17 and 26 °C. **C**, **D** Venn diagram of up-regulated DEGs in Kos and Kas

The principal component analysis (PCA) of the RNA-sequencing (RNA-seq) data revealed principal components (PCs) 1, 2, and 3 accounted for 25.2%, 17.1%, and 10.5% of the total variability, respectively (Fig. 5A). Genotypes were the key factor affecting gene expression profile. Kas and Kos were separated by the first principal component (PC1). Compared with PC1, the treatment factor (PC2) had a smaller effect, especially on the expression levels of the two genotypes at 26 °C (Fig. 5A). According to PC3, for both genotypes, the day 2 and 4 samples were clustered together and separated from the day 0 samples. Thus, the expression of many genes was induced in the seedlings during the period between day 0 and day 2. Compared with Kas, Kos exhibited higher gene expression levels on days 2 and 4 under low temperature, indicating that Kas was more sensitive to low-temperature stress than Kos.

After the seedlings were exposed to light for 2 and 4 days, the DEGs on days 2 and 4 (relative to the corresponding expression on day 0) were detected for each genotype [Kos17-(2d/0d), Kos17-(4d/0d), Kas17(2d/0d), and Kas17-(4d/0d)]. The DEGs between Kas and Kos at various time-points [(Kas17/Kos17)-0d, (Kas17/Kos17)-2d, and (Kas17/Kos17)-4d] were also detected (Fig. 5B). A total of 4,278 DEGs (2,879 up-regulated and 1,399 down-regulated) and 4,686 DEGs (2,313 up-regulated and 2,373 down-regulated) were detected in Kas17-(2d/0d) and Kas17-(4d/0d), respectively (Fig. 5C), which was more than the 3,607 DEGs (2,079 up-regulated and 1,528 down-regulated) and 4,508 DEGs (2,399 up-regulated and 2,109 down-regulated) detected in Kos17-(2d/0d) and Kos17-(4d/0d), respectively (Fig. 5B).

**Fig. 6:**
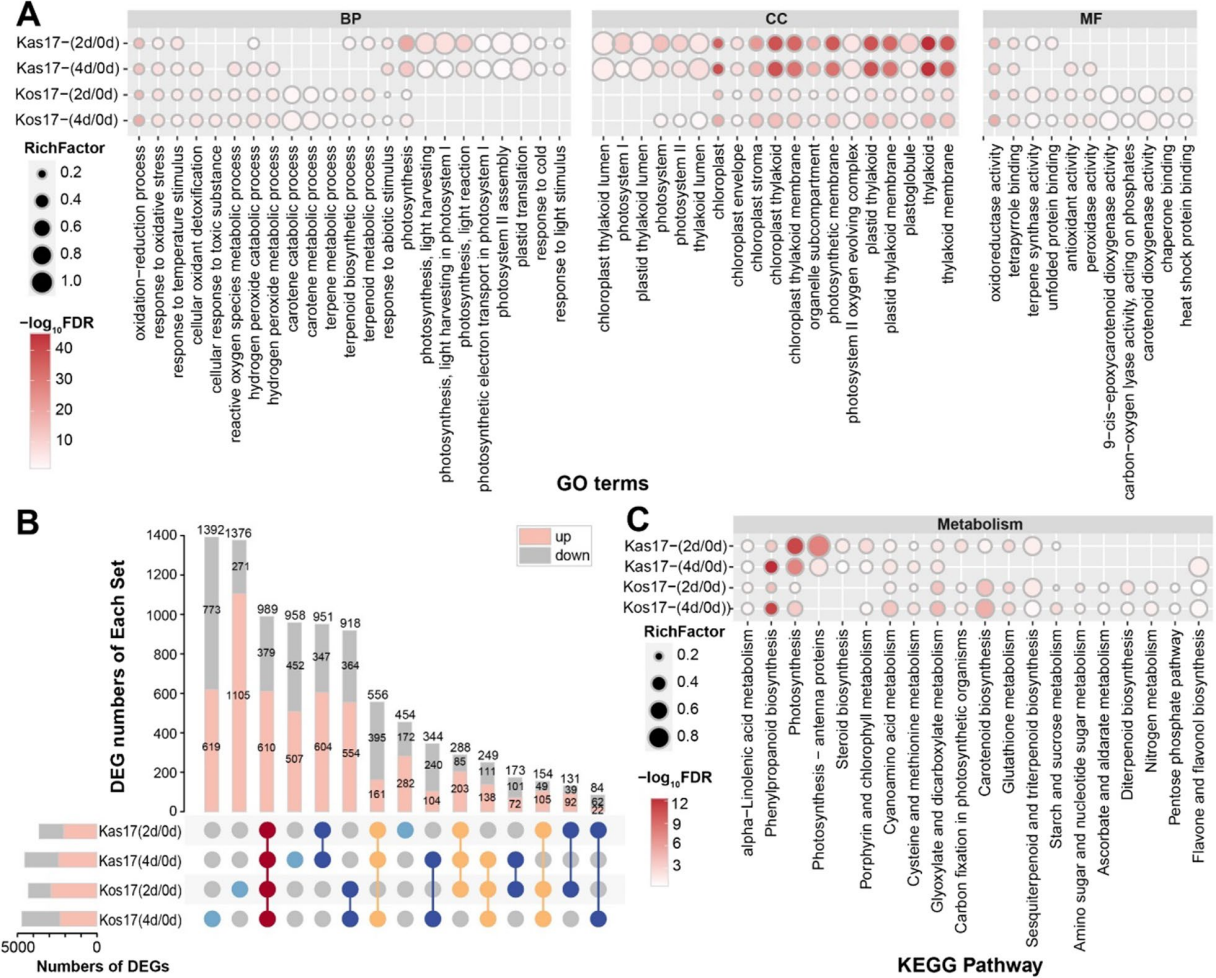
GO and KEGG enrichment analysis of DEGs. **A** Important GO terms for DEGs in Kos (compared to Kas). BP, biological process; CC, cellular component; MF, molecular function. **B** Gene expression upsets in different sets of DEGs. **C** KEGG Pathway of DEGs up-regulated in expression in Kos (compared to Kas)

The analysis of the up- and down-regulated DEGs among different sets detected 951 DEGs in both Kas17-(2d/0d) and Kas17-(4d/0d), of which 604 genes were up-regulated and 347 genes were down-regulated (Fig. 6B). There were 918 common DEGs in Kos17-(2d/0d) and Kos17-(4d/0d), among which 554 genes were up-regulated and 354 genes were down-regulated. Additionally, 989 genes were detected as DEGs in Kas17-(2d/0d), Kas17-(4d/0d), Kos17-(2d/0d), and Kos17-(4d/0d), of which 610 genes were up-regulated and 379 genes were down-regulated (Fig. 5D).

The GO and KEGG enrichment analyses of the DEGs revealed differences in the molecular mechanisms affected by low-temperature stress between the two genotypes (Fig. 6A, C). The DEGs were classified in three main GO categories (i.e., BP, CC, and MF) (Fig. 6A). The enriched GO terms among the DEGs in both genotypes were related to oxidative stress, including oxidation-reduction process, response to oxidative stress, cellular oxidant detoxification, and hydrogen peroxide catabolic process. Photosynthesis-related GO terms were significantly enriched among the DEGs in Kas17-(2d/0d) and Kas17-(4d/0d), including photosynthesis-light harvesting, photosynthesis-light reaction, photosynthetic electron transport in PSI, and PSII assembly. However, they were not significantly enriched among the DEGs in Kos17-(2d/0d) and Kos17-(4d/0d). These results reflected the oxidative stress response involving the chloroplasts of both Kas and Kos as well as the positive changes to the photosystems in Kas.

Carotenoid-related GO terms, such as carotene catabolic process, carotene metabolic process, and carotenoid dioxygenase activity, were significantly enriched among the DEGs in Kos17-(2d/0d) and Kos17-(4d/0d), but not among the DEGs in Kas17-(2d/0d) and Kas17-(4d/0d). The following GO terms were also assigned to the DEGs in Kos17-(2d/0d) and Kos17-(4d/0d): cellular response to a toxic substance and carbon-oxygen lyase activity. KEGG analysis was performed for the common up-regulated DEGs in Kos17-(2d/0d), Kos17-(4d/0d), Kas17-(2d/0d), and Kas17-(4d/0d) (Fig. 6C). Notable enriched KEGG pathways included carotenoid biosynthesis, phenylpropanoid biosynthesis, photosynthesis, and glyoxylate and dicarboxylate metabolism. Among these pathways, carotenoid biosynthesis was significantly enriched among the DEGs in Kos17-(2d/0d) and Kos17-(4d/0d), but not among the DEGs in Kas17-(2d/0d) and Kas17-(4d/0d). Thus, carotenoid biosynthesis may be critical for the cold tolerance of rice seedlings.

**Fig. 7:**
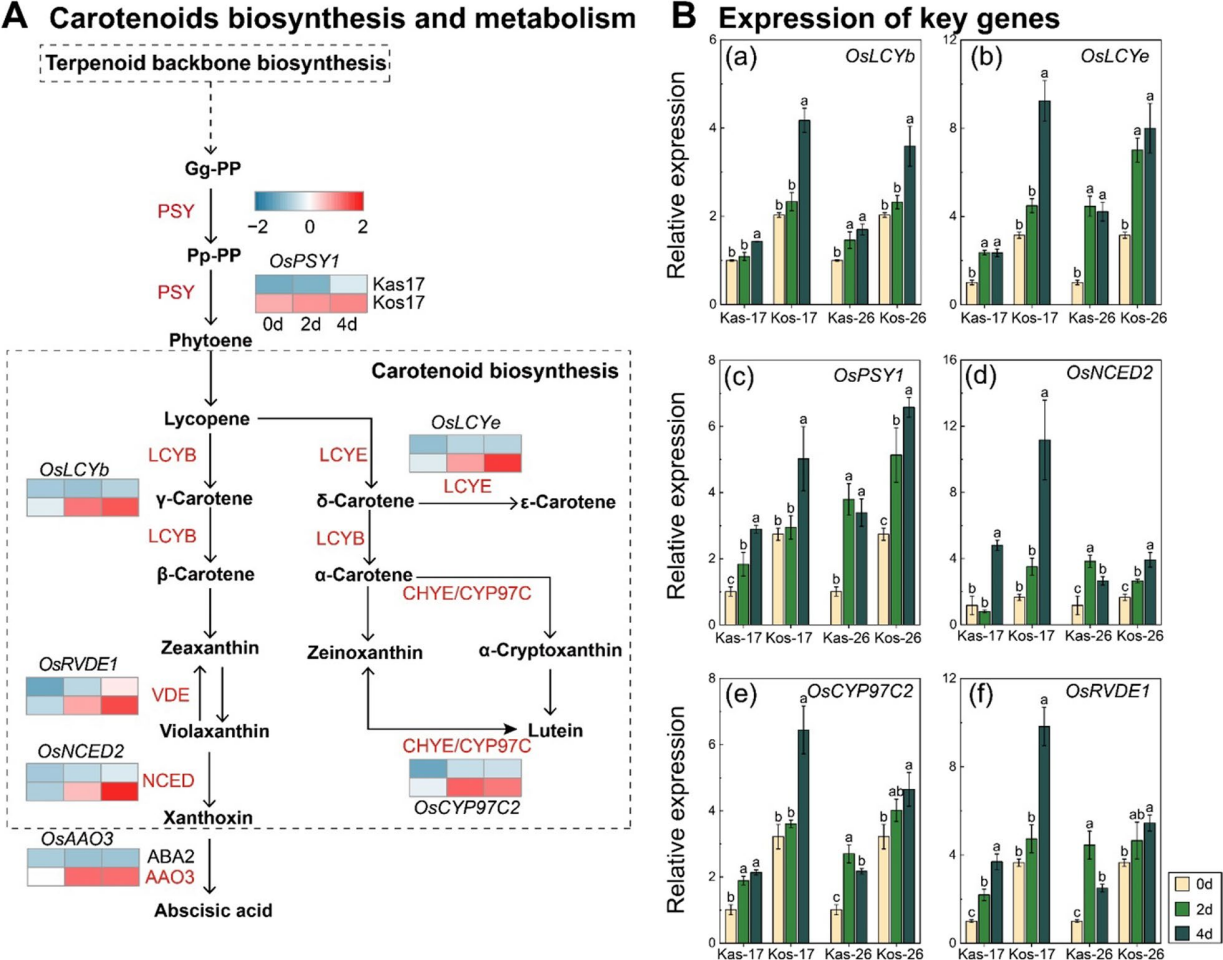
Carotenoid biosynthesis pathway and expression of carotenoid biosynthesis-related DEGs in two rice genotypes. **A** Carotenoids biosynthesis pathway and the heatmap of DEGs involved in this pathway. Detailed information on gene expression for each set of samples is available in Additional file 1: Table S3. **B** Verify the RNA-seq results of carotenoids biosynthesis pathway DEGs using qRT-PCR.

We also compared the expression levels of carotenoid biosynthesis-related genes between Kas and Kos, such as *OsPSY1*, *OsLCYe*, *OsLCYb*, *OsCYP97C2*, *OsRVDE1*, and *OsNCED2*, especially under the temperature of 17 °C (Fig. 7). The Kos cultivars showed a more significant increase in gene expression of *OsLCYe* and *OsLCYb* with increasing time of cold temperature than Kas. More specifically, the gene expression of *OsLCYe* and *OsLCYb* was elevated by 197.29% and 59.74%, respectively, on day 4 of Kos-17 compared to day 0 of light exposure. The expression level of *OsPSY1* also was higher in Kos compared to that in Kas. Meanwhile, the expression of important genes related to carotenoid biosynthesis and metabolism, such as *OsRVDE1*, *OsNCED2*, and *OsCYP97C2*, was also found higher in Kos compared to Kas, especially under low-temperature treatment. These results suggested that carotenoid biosynthesis was a key pathway to enhance cold stress in leaves under chilling stress.

### Carotenoid Biosynthesis is Critical for Conferring Cold Tolerance

The activities of the key enzymes mediating carotenoid biosynthesis were examined. After a 2-day treatment at 17 °C, there were significant increases in the PSY, LCYB, VDE, CH, and NCED activities in Kos. In contrast, there were no significant increase in the PSY, LCYB, VDE, CH, and NCED activities in Kas. More specifically, the LCYB, PSY, VDE, CH, and NCED activities in Kos at 17 °C were 2.99-fold, 1.62-fold, 2.02-fold, 2.34-fold, and 1.59-fold higher on day 2 than on day 0, respectively. However, in Kas at 17 °C, the LCYB, PSY, VDE, and NCED activities were only 0.73-fold, 0.81-fold, 0.50-fold, and 1.59-fold higher on day 2 than on day 0, respectively. It is evident that the rate of increase in the activities of key enzymes under chilling stress in Kos were significantly higher than those of Kas.

**Fig. 8:**
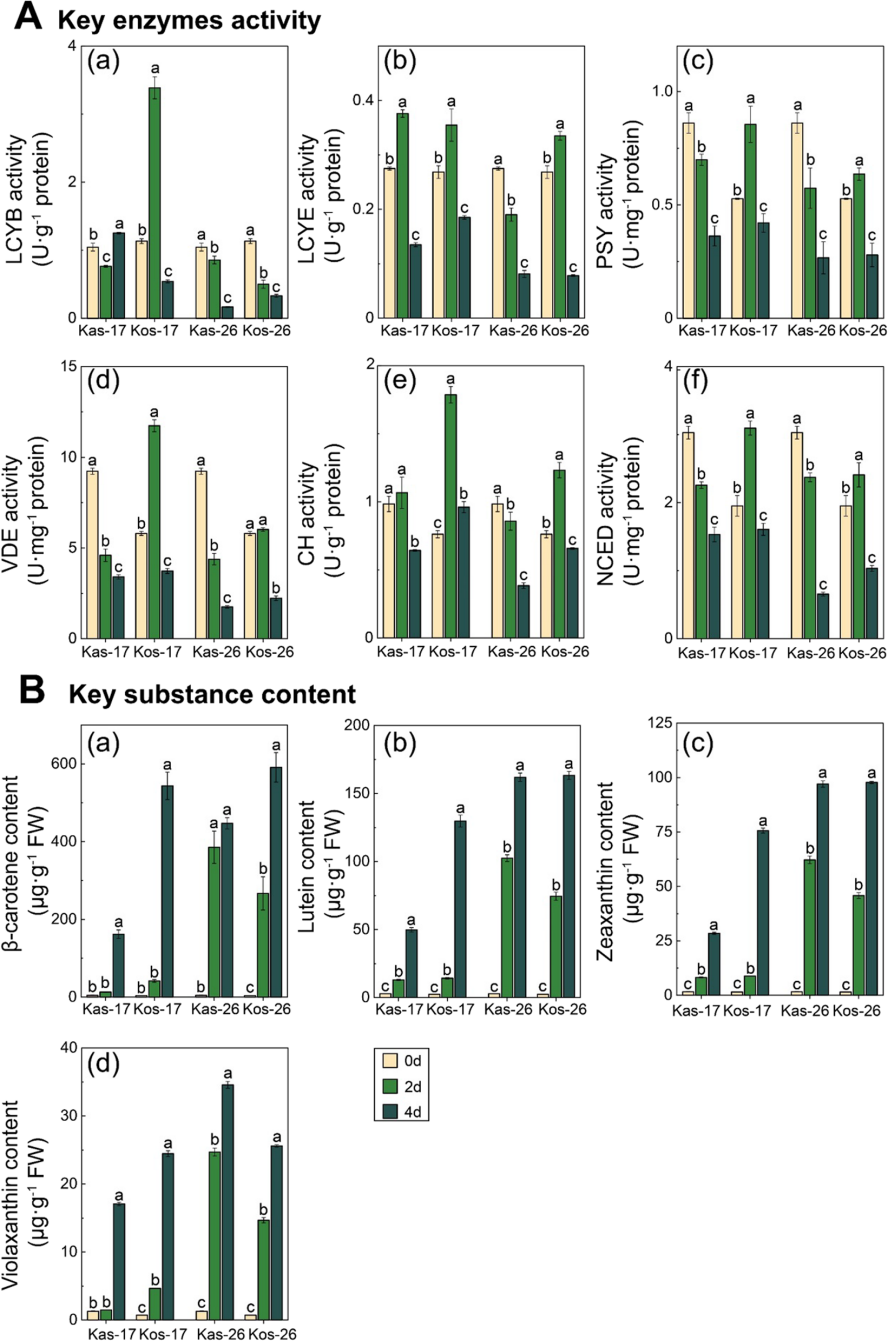
The activities of key enzyme in carotenoid biosynthesis pathway and contents of key substance in two rice genotypes during greening. **A** The activities of key enzyme in the carotenoid biosynthesis pathway, including lycopene *ε*-cyclase (LCYE), lycopene *β*-cyclase (LCYB), violaxanthin de-epoxidase (VDE), 9-*cis*-epoxy carotenoid dioxygenases (NCED), carotenoid hydroxylase (CH) and phytoene synthase (PSY). **B** Contents of key substance in the carotenoid biosynthesis pathway, including *β*-carotene, lutein, zeaxanthin, and violaxanthin

**Fig. 9:**
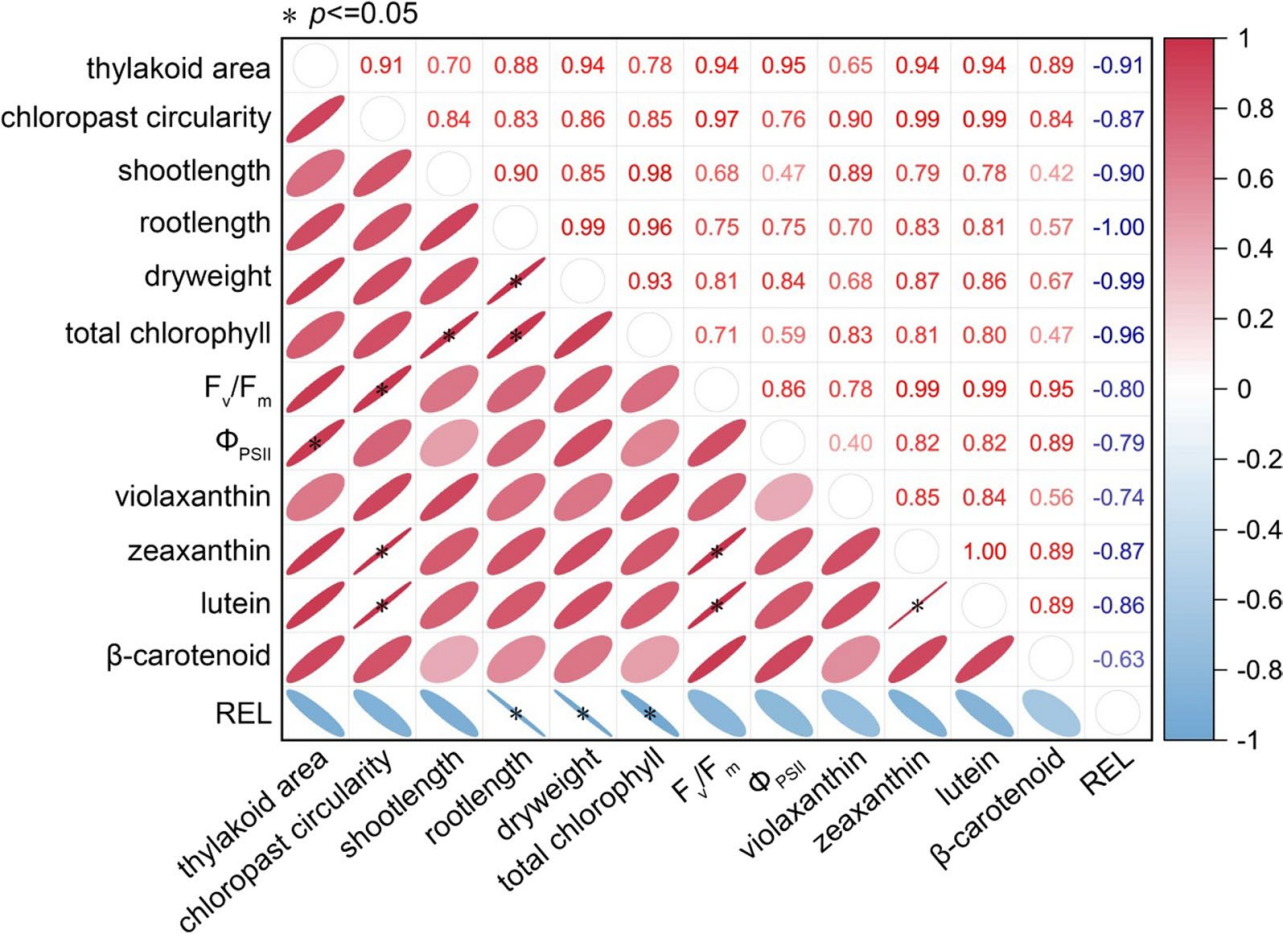
Correlation between different indicators during greening in two rice genotypes under 17 and 26 °C. Slimmer and darker color of ellipse represents stronger correlation. The asterisks (*) indicate significant differences at *p* < 0.05. Detailed data and quantification methods for the indicators of thylakoid area per chloroplast and chloroplast circularity are described in Additional file 1: Fig. S1

The contents of *β*-carotene, lutein, zeaxanthin and violaxanthin were significantly higher in Kos than in Kas on day 4 of the incubation at 17 °C, which were 3.36-fold, 2.61-fold, 2.66-fold, and 1.43-fold higher in Kos than in Kas at 17 °C, respectively. However, at 26 °C, they were only 1.32-fold, 1.01-fold, 1.00-fold, and 0.74-fold higher in Kos than in Kas, respectively (Fig. 8B; Table 1). Further, person correlation analysis was performed on the differential physiological indicators and key substance contents. The significant positive correlation was found between indicators of chloroplast and thylakoids development (i.e., thylakoid area, chloroplast circularity, F_v_/F_m_, and Φ_PSII_) and carotenoid content (i.e., lutein and zeaxanthin) (Fig. 9). These results suggest that carotenoids contribute to the enhancement of response to low-temperature stress.

## Discussion

Rice is a thermophilic crop sensitive to low temperatures (Mackill and Lei 1997; Fukuda and Terao 2015; Fukuda et al. 2021). Rice seedling leaves become chlorotic and appear white under cold conditions, especially during the greening process (Yoshida et al. 1996; Fukuda et al. 2021). After the exposure to light at 17 °C, Kas leaves were yellow as they failed to turn green in the study. However, the Kos seedlings incubated under chilling stress underwent a normal greening process, resulting in green leaves (Fig. 1). Furthermore, in response to low-temperature stress, the Kos leaves showed relatively normal oval chloroplasts and more thylakoids than the Kas leaves. Chlorophyll levels and F_v_/F_m_ values are important indicators of rice’s tolerance to low temperature stress (Zhao et al. 2020). Chlorophyll content, F_v_/F_m_ and Φ_PSII_ values indicated a significant increase for Kos under low temperature, which enable the plant to maintain its photosynthetic characteristics, thereby improving cold tolerance.

Carotenoids, as photosynthetic pigments, could protect chlorophylls from adverse factors in plants (Maslova et al. 2021). Kayess et al. (2020) reported Boro rice seedling (BR-16) with higher total carotenoid content exhibited comparatively better potentiality to survive at low temperatures (below 15˚C). Besides, low-temperature stress changed the accumulation of carotenoids in *Citrus sinensis* (Tao et al. 2012), *Camellia sinensis* (Yang et al. 2021), *Capsicum annuum* (Li et al. 2021), and *Osmanthus fragrans* (Wang et al. 2022). In this study, there was a significant increase in the content of carotenoids (i.e., *β*-carotene, lutein, and zeaxanthin) in Kos during cold stress, and suggested the carotenoid biosynthesis pathway was active in Kos response to chilling stress. In the carotenoid biosynthesis, PSY, acted as the first key enzyme, governed carotenoid pool size (Nisar et al. 2015). Overexpression of *PSYs* enhanced total carotenoid contents and substantially increased synthesis of *β*-carotene in the rice callus (Paine et al. 2005). The expression of *OsPSY1*, *OsPSY2*, and *OsPSY3* were significantly increased in rice seedlings subjected to cold exposure (Pan et al. 2022), and also the up-regulation of *CsPSY1* expression in tea plant at low temperature was reported (Yang et al. 2021). LCYB was an essential enzyme that catalyzes the conversion of lycopene into *α*-carotene and *β*-carotene in carotenoid biosynthesis pathway (Cazzonelli et al. 2009; Kang et al. 2018; Wang et al. 2021c). The overexpression of *IbLCYB2* increased synthesis of *α*-carotene, *β*-carotene, lutein, zeaxanthin and enhance tolerance to salt, drought and oxidative stresses in sweet potato (Kang et al. 2018). The transcript levels of *OsPSY1*, *OsLCYb* and the activities of PSY, LCYB were up-regulated in Kos at low temperature, which maybe a manifestation of enhancing low temperature tolerance of Kos.

(Havaux et al. 2007; Havaux 2014; Nisar et al. 2015)The xanthophyll cycle was formed by the mutual transformation of violaxanthin, antheraxanthin and zeaxanthin under the catalysis of ZEP and VDE, which protected the normal photosynthesis of plants under various stress such as extreme temperature (Hao et al. 2022; Pan et al. 2022). Zeaxanthin could eliminate oxidative substances, which was a potent low-molecular-weight antioxidant promoted by the enzyme of VDE (Jahns and Holzwarth 2012; Leonelli et al. 2017; Wang et al. 2021b). Lutein was the most abundant carotenoid pigment in photosynthetic tissues of plants, which was mainly composed of two heme-containing cytochrome P450 type hydroxylases (CYP97A and CYP97C) that catalyze the synthesis of *β*- and *ε*- cyclic hydroxylation of α-carotene (Dall’Osto et al. 2007; Jahns and Holzwarth 2012; Nisar et al. 2015). The transcript levels of *OsRVDE1* and *OsCYP97C2* were significantly increased at low temperatures in Kos. As a result, zeaxanthin and lutein were accumulated in Kos during cold stress, and may help alleviate the detrimental effects of chilling stress on chloroplasts, thereby promoting the leaf greening process.

In our study, we observed the relationship between the expression of *OsPSY1*, *OsLCYe*, *OsRVDE1*, *OsCYP97C2*, and *OsNCED2* and the activities of PSY, LCYE, VDE, CH, and NCED, was not entirely consistent. Firstly, many of these enzyme families are consisted of multiple gene members, such as *OsPSY1*, *2* and *3* in *PSY* family (Welsch et al. 2008), *CYP97A*, *B*, *and C* in *CYP97* gene subfamily (Bak et al. 2011), and *OsNCED1*, *2*, *3*, *4*, and *5* in *NCED* gene subfamily (Changan et al. 2018). Therefore, changes in the expression of a gene are not representative of changes in the enzyme family. Additionally, post-translational modifications (PTMs) may play a role in this variability. It’s worth noting that PTMs can be induced by carotenoid-derived aldehydes (CDA), potentially leading to cellular toxicity due to the excessive accumulation of carotenoids (Kalariya et al. 2011); Chen et al. 2022). It was also reported that PSY activity can be regulated through ubiquitination, a distinct form of PTM, resulting in degradation facilitated by plastid protein sensing RING E3 ligase 1 (Wang et al. 2020). Hence, we conducted the prediction of potential PTM sites and their confidence scores in the protein sequences of *OsPSY1*, *OsRVDE1*, *OsCYP97C2*, and *OsNCED2* using MusiteDeep analysis (Additional file 2: Table S4), with reference to a previous study (Chen et al. 2022). Phosphorylation, hydroxylation, and ubiquitination were PTM types with higher confidence and frequent occurrences. While these enzyme activity results may not align with the gene expression results, they still indicate an increasing trend of carotenoid-related enzymes in Kos under low-temperature conditions, along with a significant decrease in Kas. Nonetheless, the underlying mechanisms and significance of PTMs in carotenoid biosynthesis remain a subject for future investigation. Further research, such as proteomic or MeRIP-seq studies, may be warranted in the future.

## Conclusions

In conclusion, comparative transcriptome and morphophysiological analyses were conducted for Kos and Kas, which had distinct greening phenotypes under low-temperature condition. The Kos seedlings underwent a normal greening process at 17 °C and contained more chlorophyll and better developed chloroplasts than Kas, indicative of the greater chilling stress tolerance of Kos. The significant up-regulation of the expression of carotenoid biosynthesis-related genes in Kos is suggestive of the positive role of carotenoids in the greening process at low temperatures. The expression levels of the key genes involved in carotenoid biosynthesis, such as *OsLCYb*, *OsPSY1*, and *OsRVDE1* were up-regulated in Kos. The increased PSY, and LCYB, activities as well as the accumulation of *β*-carotene, lutein, and zeaxanthin in Kos indicate carotenoid biosynthesis is crucial for preventing leaf chlorosis in Kos at low temperatures. This study provides valuable insights into the physiological and molecular mechanisms mediating the adaptation of rice to low temperatures, which may be useful for developing strategies for improving rice seedling quality.

## Materials and Methods

### Plant Materials and Experimental Design

The cold-tolerant rice cultivar Koshihikari (Kos; *Oryza sativa* L., *japonica*) and the cold-susceptible cultivar Kasalath (Kas; *Oryza sativa* L., *indica*) were obtained from the China National Rice Research Institute. Healthy and intact seeds were surface-sterilized in 30% (v/v) H_2_O_2_ for 20 min. They were subsequently immersed in distilled water for 24 h and then incubated in darkness for 8 h on damp filter paper at 32 °C. The seeds were manually transferred to a carbon-based composite seedling substrate specifically designed for rice in standard rice transplanter seedling trays, with each tray containing 90 g germinated seeds. The trays were moved into incubators with different temperatures when 80% of the seeds had germinated and the buds were approximately 10 mm long. The control group was incubated at 26 °C (suitable growth temperature) with a light intensity of 120 µmol m^−2^ s^−1^, whereas the low-temperature group was incubated at 17 °C with a light intensity of 120 µmol m^−2^ s^−1^. Seedling samples were collected on days 0, 2, and 4 for the analysis of various indices. Specifically, the shoot length, root length, and shoot weight of the Kas and Kos seedlings were measured, with three replicates per treatment, each comprising at least 30 randomly selected seedlings. Another sample of seedlings underwent a tap water wash and several distilled water rinses. These samples were drained, then kept at − 80 °C for the estimation of additional parameters.

### Determination of the Carotenoid and Chlorophyll Contents and Relative Electrolyte Leakage

Fresh leaves were collected from rice seedlings after 0, 2, and 4 days of the temperature treatments. The leaves were placed in a darkened container and soaked in 95% ethanol until they turned white. Subsequently, the absorbance of the solution at 470, 649, and 665 nm was determined using the SPECORD 200 spectrophotometer (Analytik, Jena, Germany). The chlorophyll *a* and *b* contents were calculated as previously described (Song et al. 2023).

Relative electrolyte leakage (REL) was analyzed using a published method (Bajji et al. 2002). Briefly, fresh leaves were cleaned with deionized water, cut into pieces, and then weighed before being added to tubes containing deionized water. The leaves were vacuum desiccated for 1 h before being placed at room temperature for 4 h. Throughout this period, the leaf samples were intermittently shaken. A conductivity meter (Mettler Toledo, Zurich, Switzerland) was used to determine the conductivity of the extraction solution.

The contents of key substances in the carotenoid biosynthesis, including *β*-carotene, lutein, zeaxanthin, and violaxanthin, were quantified as previously mentioned (Ma et al. 2018; Yang et al. 2021). To extract compounds, acetone was added to fresh rice leaves (200 mg) prior to a 5-min sonication. The extraction using acetone was repeated multiple times. The resulting extracts were mixed with 1 mL acetone, filtered using a syringe filter, and assessed with an ultra-high-performance liquid chromatography (UHPLC) system. The UltiMate 3000 UHPLC system (Thermo Scientific Dionex, Sunnyvale, CA, USA) was employed to determine the *β*-carotene, lutein, zeaxanthin, and violaxanthin contents. The UHPLC separation and characterization were completed using the Compass C18 reversed-phase column (250 mm × 4.6 mm, 5 μm). The column temperature was 30 °C and the walk-away time was 20 min. A Finnigan Surveyor PDA Plus Detector (Thermo, Hennigsdorf, Germany) was used to measure the UV-vis absorbance between 210 and 600 nm.

### Chlorophyll Fluorescence Assay and Examination of the Chloroplast Ultrastructure

The chlorophyll fluorescence of the rice seedlings was analyzed after a 4-day treatment at 17 or 26 °C. Before the assay, the seedlings were placed in a darkened container for at least 20 min (dark adaptation), after which the IMAGING-PAM Multi-function Modulated Fluorescence Imaging System (Walz, Bavaria, Germany) was used to determine the fluorescence kinetic profile of the seedlings. The ImagingWinGige software was used to record and extract the following photosystem II (PSII) parameters: initial chlorophyll fluorescence (F_o_) in dark-adapted leaves, maximum fluorescence (F_m_) in dark-adapted leaves, maximum fluorescence (F_m_′) in the light-adapted state, steady-state chlorophyll fluorescence (F_s_) in the light-adapted state, effective quantum yield of PSII [i.e., Φ_PSII_ = (F_m_′ − F_s_)/F_m_′], and potential maximum photosynthetic capacity of PSII [i.e., F_v_/F_m_ = (F_m_ − F_o_)/F_m_]. To reduce the effect of external light, the measuring head was clothed with a black cover for all processes.

Chloroplast ultrastructural development in rice leaves was examined using a transmission electron microscope. Fresh leaf tissues (volume less than 1 mm^3^) were rapidly fixed in a 2.5% glutaraldehyde solution under vacuum conditions and then rested at 20 °C for 2 h. They were then incubated at 4 °C. The samples were rinsed three times with 0.1 M phosphate-buffered saline (PBS) (pH 7.4) for 15 min and then fixed in a solution consisting of 1% osmium acid and 0.1 M PBS (pH 7.4) for 5 h at 20 °C. The samples were rinsed three times with 0.1 M PBS (pH 7.4) for 15 min before and after fixation, and then were dehydrated with different gradients of ethanol (30%, 50%, 70%, 80%, 90%, and 100%). Pure 812 embedding agent (SPI-Pon 812; Structure Probe, Inc., USA) was poured into embedding plates, after which the samples were added to the plates and dried overnight in an oven at 37 °C before being polymerized in the oven at 60 °C for 48 h. The samples were sliced (60–80 nm sections) using an ultrathin slicer (Leica Microsystems, Ltd., Wetzlar, Germany) and then stained for 15 min using a 2% uranyl acetate saturated alcohol solution and lead citrate. After drying overnight at room temperature, the chloroplast ultrastructure was examined and images were captured using the HT7700 transmission electron microscope (Hitachi, Tokyo, Japan).

### RNA Extraction and Transcriptome Sequencing

Total RNA was extracted from the shoots of the seedlings incubated at 26 or 17 °C for 0, 2, and 4 days using the RNeasy^®^ Mini kit (Qiagen, Hilden, Germany). Because each treatment was performed in triplicate, 30 RNA samples were prepared. The RNA quality was evaluated using 1.0% agarose gel electrophoresis and confirmed (i.e., RNA integrity number > 8.0) using the 2100 Bioanalyzer (Agilent Technologies, Santa Clara, CA, USA). The cDNA libraries were built using the NEB Next^®^ Ultra™ RNA Library Prep Kit for Illumina (NEB #E7530, USA). They were subsequently sequenced on the Illumina NovaSeq 6000 platform (Illumina Inc., CA, USA), which yielded an average of 6 billion clean reads per sample. To get rid of reads with adapters and poor-quality sequences, the raw reads were filtered. The high-quality reads were retained for the downstream analyses. To align the data, the reference genome and gene model annotation files were obtained from the Rice Genome Annotation Project (Kawahara et al. 2013; Sakai et al. 2013).

### Analysis of Differential gene Expression

For each sample, gene expression levels were determined in terms of fragments per kilobase of transcript sequence per million base pairs sequenced (FPKM) values using the RSEM software (version 1.2.15) (Li and Dewey 2011). Differentially expressed genes (DEGs) were detected using the DESeq2 software, with a false discovery rate < 0.01 and |log_2_(fold-change)| >1 set as the thresholds for significance (Anders and Huber 2010). The PRCOMP function of the R package (version 3.5.0) was used to perform the principal component analysis (PCA). The PCA plot was visualized using SCATTERPLOT3D. Gene Ontology (GO) and Kyoto Encyclopedia of Genes and Genomes (KEGG) analyses were performed on the unigenes with up-regulated expression levels on days 2 and 4 of the low-temperature (17 °C) treatment period (compared to the corresponding levels on day 0). The significantly enriched functional categories (Fisher’s test; *p* < 0.05) were visualized using ggplot2. After the Z-score normalization of the original RNA sequencing (RNA-seq) data, heatmaps were generated using the ‘pheatmap’ function of the R package.

### Quantitative real-time PCR

The quantitative real-time PCR (qRT-PCR) analysis was performed using the 7500 Real-Time PCR System and the SYBR Premix Ex Taq™ kit to determine the relative expression levels of six genes encoding key enzymes for carotenoid synthesis (i.e., *OsPSY1*, *OsLCYe*, *OsLCYb*, *OsCYP97C2*, *OsRVDE1*, and *OsNCED2*). An endogenous actin-encoding gene (NM 197,297) was used as the reference control for normalizing expression levels. The qRT-PCR data were analyzed using the 2^−ΔΔCt^ method (Pfaffl, 2001). For each gene, the expression level (i.e., mRNA level) for Kas at 17 °C (designated as Kas-17) was set to 1. The qRT-PCR primers are detailed in Additional file 1: Table S1.

### Assays of the Activities of Carotenoid Biosynthesis Pathway Enzymes

To quantitatively analyze enzyme a ctivities, shoots were homogenized and centrifuged. The supernatants were collected and analyzed for the activities of PSY, LCYE, LCYB, CH, VDE, and NCED using commercial enzyme-linked immunosorbent assay kits (Meimian, China) as previously described (Wu et al. 2020). Protein contents were analyzed according to the Bradford method (Bradford 1976; Jones et al. 1989).

### Statistical Analyses

Data are herein presented as the mean and standard error. At least three biological replicates were included in all experiments. The data for the seedlings incubated at 26 or 17 °C were analysed either by one-way analysis of variance (ANOVA), two-way ANOVA, or three-way ANOVA depending on the experiment using SPSS version 25.0 (SPSS, Chicago, IL, USA). The figures were made using Origin 9.1 (version 9.1) (Origin Lab, Northampton, MA, USA).

### Supplementary Information

**Additional file 1: Table S1.** Primers used for qRT-PCR analysis. **Table S2.** Overview of transcriptome sequencing. **Table S3**. Gene expression in the heat map for each set of samples. **Figure S1** Chloroplast circularity and thylakoid membrane area of two rice genotypes at 17 and 26 °C on day 4. **a** Average circularity of chloroplasts of two rice genotypes. **b** Average area of thylakoid membrane per chloroplast of two rice genotypes. **Indicate significant differences at *p* < 0.01 probability levels.

**Additional file 2: Table S4.** Predicted PTM sites of *OsPSY1*, *OsLCYe*, *OsCYP97C2*, *OsRVDE1*, and *OsNCED2.*

## Data Availability

The raw data files used for transcriptomic analysis have been uploaded to the National Center for Biotechnology Information (NCBI) under accession number PRJNA1028983. The datasets supporting the conclusions of this article are included within the article and its additional files.

## Abbreviations

BCHs: *β*-Ring hydroxylases
CHs: Carotenoid hydroxylases
DEGs: Differentially expressed genes
GO: Gene Ontology
KEGG: Kyoto Encyclopedia of Genes and Genomes
LCYB: Lycopene *β*-cyclase
LCYE: Lycopene *ε*-cyclase
NCED: 9-*Cis*-epoxycarotenoid dioxygenase
PAM: Pulsed amplitude modulation
PCA: Principal component analysis
PSY: Phytoene synthase
REL: Relative electrolyte leakage
ROS: Reactive oxygen species
VDE: Violaxanthin de-epoxidase
ZEP: Zeaxanthin epoxidase
