# Supplementary material for: Comparative Transcriptome Combined with Morphophysiological Analyses Revealed Carotenoid Biosynthesis for Differential Chilling Tolerance in Two Contrasting Rice (Oryza sativa L.) Genotypes
Source: Rice (N Y). 2023 Nov 25;16:52. doi: 10.1186/s12284-023-00669-6 (PMC10676345; doi:10.1186/s12284-023-00669-6)
Supplement: Supplementary file 1 — Additional file 1: Table S1. Primers used for qRT-PCR analysis. Table S2. Overview of transcriptome sequencing. Table S3. Gene expression in the heat map for each set of samples. Figure S1 Chloroplast circularity and thylakoid membrane area of two rice genotypes at 17 and 26 °C on day 4. a Average circularity of chloroplasts of two rice genotypes. b Average area of thylakoid membrane per chloroplast of two rice genotypes. **Indicate significant differences at p < 0.01 probability levels. [file 12284_2023_669_MOESM1_ESM.docx]

**Supplementary Material**

**Table S1 Primers used for qRT-PCR analysis**

|  | **Gene name** | **Forward primer** | **Reverse primer** |
| --- | --- | --- | --- |
| Os01g0581300 | *OsLCYe* | ATTCAAAGACCTGGGCCTGG | GCATCGTCTCAGCAACTCCT |
| Os10g0546600 | *OsCYP97C2* | cttgcTAGCCGTGAAGAGGT | AACACGGTCAACCTCTGCTT |
| Os12g0435200 | *OsNCED2* | GAGCCTTGAGTTCGGTGTCA | CAGCAAAGCACCCTAGACCA |
| Os02g0190600 | *OsLCYb* | TGTCGTCGAGGCTCTTCTTG | CTGGATGAGGTTGCCGATCA |
| Os04g0379700 | *OsRVDE1* | GGCCTCAATCCCACTTTCGA | TCCTGCACAAACCGCTGTAT |
| Os06g0729000 | *OsPSY1* | TCCTCGGAGCAGAAGGTGTA | GTCTTGGCATACTCCTCGCA |
|  | *actin* | TTATGGTTGGGATGGGACA | AGCACGGCTTGAATAGCG |

**Table S2 Overview of transcriptome sequencing**

| Sample | Raw Reads | Clean reads | Clean Data(Gb) | Q20(%) | Q30(%) |
| --- | --- | --- | --- | --- | --- |
| Kas_0d_1 | 53037694 | 52926338 | 7.91 | 97.7 | 93.95 |
| Kas_0d_2 | 52952330 | 52699790 | 7.86 | 97.49 | 93.53 |
| Kas_0d_3 | 49256200 | 49139174 | 7.35 | 97.4 | 93.33 |
| Kos_0d_1 | 54055218 | 53916260 | 8.05 | 97.55 | 93.72 |
| Kos_0d_2 | 55873832 | 55728752 | 8.33 | 97.63 | 93.82 |
| Kos_0d_3 | 45646408 | 45521390 | 6.81 | 97.45 | 93.43 |
| Kas26_2d_1 | 50485942 | 50387200 | 7.53 | 97.87 | 94.31 |
| Kas26_2d_2 | 44077606 | 43977892 | 6.57 | 97.6 | 93.72 |
| Kas26_2d_3 | 53709008 | 53545138 | 8.00 | 97.49 | 93.73 |
| Kos26_2d_1 | 48534788 | 48409824 | 7.21 | 97.75 | 94.3 |
| Kos26_2d_2 | 49544154 | 49362822 | 7.07 | 97.53 | 93.99 |
| Kos26_2d_3 | 47940784 | 47811462 | 7.14 | 97.73 | 94.15 |
| Kas17_2d_1 | 49644708 | 49530774 | 7.41 | 97.37 | 93.21 |
| Kas17_2d_2 | 51265800 | 51144904 | 7.65 | 97.45 | 93.43 |
| Kas17_2d_3 | 49280226 | 49143274 | 7.35 | 97.62 | 94 |
| Kos17_2d_1 | 54144548 | 53840928 | 8.05 | 96.41 | 91.14 |
| Kos17_2d_2 | 49100576 | 48979502 | 7.33 | 97.54 | 93.64 |
| Kos17_2d_3 | 45047512 | 44929960 | 6.72 | 97.58 | 93.76 |
| Kas26_4d_1 | 53514002 | 53371868 | 7.99 | 97.52 | 93.64 |
| Kas26_4d_2 | 48045992 | 47909890 | 7.17 | 97.42 | 93.42 |
| Kas26_4d_3 | 49208394 | 49085412 | 7.35 | 97.4 | 93.31 |
| Kos26_4d_1 | 48130536 | 48010622 | 7.18 | 97.47 | 93.57 |
| Kos26_4d_2 | 50902434 | 50568962 | 7.20 | 97.29 | 93.68 |
| Kos26_4d_3 | 56676756 | 56485312 | 8.41 | 97.51 | 93.85 |
| Kas17_4d_1 | 48396154 | 48303414 | 7.22 | 97.59 | 93.73 |
| Kas17_4d_2 | 56629718 | 56423548 | 8.44 | 97.32 | 93.25 |
| Kas17_4d_3 | 51187940 | 50965264 | 7.62 | 97.18 | 92.95 |
| Kos17_4d_1 | 49892602 | 49759610 | 7.45 | 97.29 | 92.99 |
| Kos17_4d_2 | 47747004 | 47609242 | 7.13 | 97.23 | 92.9 |
| Kos17_4d_3 | 50561738 | 50435092 | 7.55 | 97.56 | 93.63 |

**Table S3 Gene expression in the heat map for each set of samples**

| Name | Gene number | Kas_0d | Kas17_2d | Kas17_4d | Kos_0d | Kos17_2d | Kos17_4d |
| --- | --- | --- | --- | --- | --- | --- | --- |
| *OsLCYe* | Os01g0581300 | 3.24 | 7.41 | 5.08 | 2.15 | 15.22 | 10.01 |
| *OsCCD8a* | Os01g0566500 | 0.06 | 0.50 | 1.06 | 2.40 | 17.30 | 19.98 |
| *OsCYP97C2* | Os10g0546600 | 0.36 | 0.29 | 0.63 | 0.72 | 1.23 | 1.57 |
| *OsABA8OX3* | Os09g0457100 | 5.37 | 22.35 | 21.00 | 22.56 | 88.95 | 123.23 |
| *OsNCED2* | Os12g0435200 | 0.05 | 0.00 | 0.37 | 0.00 | 2.91 | 17.99 |
| *OsAAO3* | Os07g0282300 | 2.90 | 2.32 | 3.17 | 14.41 | 5.00 | 7.89 |
| *OsLCYb* | Os02g0190600 | 0.00 | 0.00 | 0.00 | 3.18 | 5.53 | 5.14 |
| *OsRVDE1* | Os04g0379700 | 1.37 | 1.19 | 0.71 | 10.85 | 6.87 | 6.33 |
| *OsABA8OX1* | Os02g0703600 | 0.24 | 0.13 | 0.10 | 1.18 | 0.94 | 1.08 |
| *D27* | Os11g0587000 | 1.32 | 0.28 | 0.05 | 2.84 | 3.32 | 5.89 |
| *OsPSY1* | Os06g0729000 | 23.18 | 15.76 | 25.89 | 60.16 | 56.93 | 59.32 |
| *OsLCYe* | Os01g0581300 | 1.94 | 2.71 | 1.55 | 12.28 | 9.35 | 5.36 |
| *OsCCD8a* | Os01g0566500 | 1.40 | 1.59 | 1.58 | 5.97 | 5.54 | 3.73 |
| *CYP97C2* | Os10g0546600 | 0.00 | 0.02 | 0.00 | 0.80 | 0.66 | 0.65 |
| *OsABA8OX3* | Os09g0457100 | 0.18 | 0.16 | 0.02 | 1.79 | 2.93 | 3.83 |
| *OsNCED2* | Os12g0435200 | 21.47 | 37.04 | 25.18 | 76.48 | 98.90 | 55.92 |
| *OsAAO3* | Os07g0282300 | 101.97 | 293.88 | 189.83 | 643.15 | 801.08 | 953.56 |
| *OsLCYb* | Os02g0190600 | 23.62 | 281.87 | 124.30 | 266.21 | 1374.67 | 1655.90 |


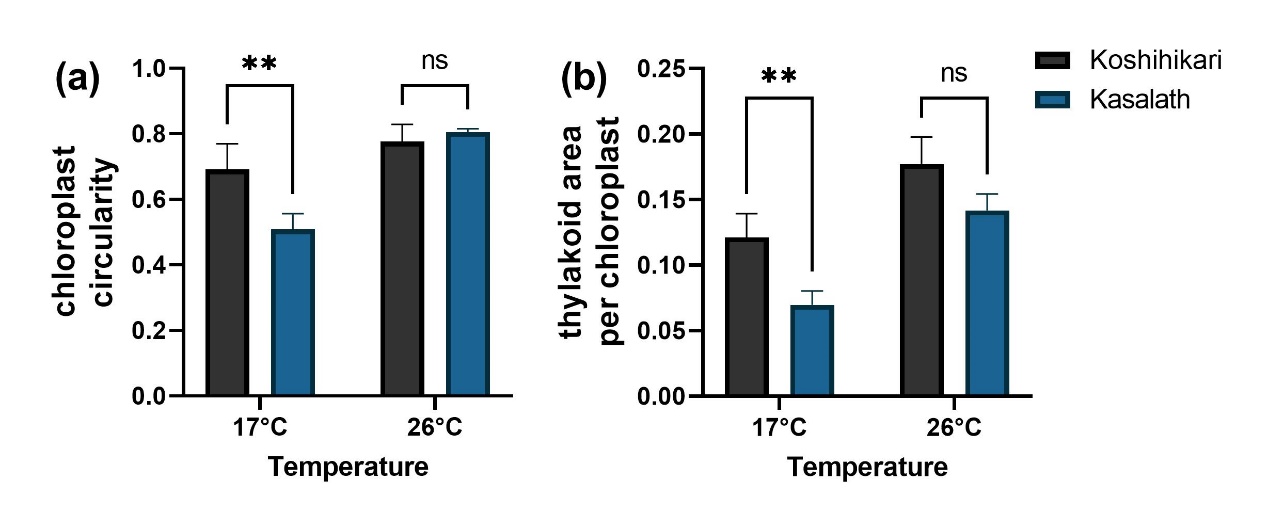


**Figure S1** Chloroplast circularity and thylakoid membrane area of two rice genotypes at 17 and 26 °C on day 4. (a) Average circularity of chloroplasts of two rice genotypes. (b) Average area of thylakoid membrane per chloroplast of two rice genotypes. ** indicate significant differences at *p*<0.01 probability levels.

Chloroplast circularity and thylakoid membrane area per chloroplast were quantified following the methodology established in prior research (Alamdari et al. 2020; Grigorova et al. 2012). ImageJ was employed as the quantitative analysis software for transmission electron microscopy images. For each genotype and temperature treatment, parameters were collected from at least three different cells chosen from at least three different seedlings. The quantified chloroplast circularity and thylakoid membrane area parameters were subsequently utilized in a multivariate correlation analysis within this study, with reference to a previous study (Song et al. 2022).

**Reference**

Alamdari K, Fisher KE, Sinson AB, Chory J, Woodson JD (2020) Roles for the chloroplast-localized pentatricopeptide repeat protein 30 and the 'mitochondrial' transcription termination factor 9 in chloroplast quality control. Plant J. 104:735-751

Grigorova B, Vassileva V, Klimchuk D, Vaseva I, Demirevska K, Feller U (2012) Drought, high temperature, and their combination affect ultrastructure of chloroplasts and mitochondria in wheat (*Triticum aestivum L*.) leaves. J. Plant Interact. 7:204-213

Song SY, Song SY, Nian PW et al. (2022) Transcriptomic Analysis Suggests a Coordinated Regulation of Carotenoid Metabolism in Ripening Chili Pepper (*Capsicum annuum* var. *conoides*) Fruits. Antioxidants 11:11
